# *C. elegans*: A potent model for high-throughput screening experiments investigating the FLASH effect

**DOI:** 10.1016/j.ctro.2023.100712

**Published:** 2023-12-09

**Authors:** Lucas Schoenauen, François-Xavier Stubbe, Dirk Van Gestel, Sébastien Penninckx, Anne-Catherine Heuskin

**Affiliations:** a NAmur Research Insitute for Life Sciences, University of Namur, Belgium; b Department of Radiation Oncology, Institut Jules Bordet, Hopital Universitaire de Bruxelles (HUB), Université Libre de Bruxelles, Brussels, Belgium

**Keywords:** Proton flash, Electron flash, C. elegans embryo

## Abstract

This study explores the effects of UHDR irradiation on *Caenorhabditis elegans e*mbryos. UHDR proton and electron beams demonstrate a sparing effect, aligning with literature findings. This highlights *C. elegans *suitability as a screening model for studying the LET impact on the FLASH effect, reinforcing its potential in radiation research.

## Highlights

• Conventional irradiation using X-rays, protons and electrons induces a growth delay in *Caenorhabditis elegans.*
• Ultra-high dose rate irradiation using protons and electrons has a smaller effect on the growth delay than conventional.
• This is the first study to highlight the FLASH effect in *C. elegan*s.
• *C. elegans* can be used as a model to investigate the FLASH effect.

## 1 Introduction

Radiotherapy is considered a fundamental component of cancer treatment modalities, as it can be used for curative and palliative purposes. Although the last few decades have seen tremendous progress in dose conformation, enabling ionizing radiation to target the tumor site very precisely, modern radiotherapy is still limited by the induction of adverse events in exposed healthy tissue. In this context, FLASH radiotherapy (FLASH-RT) is emerging as the next revolution in the field, typically delivering the prescribed dose within a few milliseconds [1] compared to minutes in conventional dose rates (CDR) settings. Interestingly, recent studies [2], [3], [4] have shown that FLASH-RT induces less adverse events in healthy tissues than CDR radiotherapy (CONV-RT) while reaching similar tumor control. The FLASH effect has been observed at ultra-high dose rates (UHDR), usually above a threshold of 100 Gy/s (mean dose rate), although this sole condition is not sufficient to trigger the effect and a complex, and so far undefined, combination of beam parameters is required. Nowadays, this complex differential effect is only demonstrated *in vivo* [1], requiring the use of various mammal models including mice, cats, dogs and pigs. Current preclinical research calls for broad-range investigations, from a better understanding of the mechanistic aspects to the optimization of physical parameters to modulate the FLASH effect. In this context, the use of aforementioned animal models limits large-scale high-throughput investigation due to long response time (weeks to months), ethical issues and expensive/tedious maintenance. In this short communication, we address these issues by using nematode *Caenorhabditis elegans* as a suitable model.

*C. elegans*, a free-living nematode, is a microscopic (about 1 mm in length and 100 µm in diameter) worm that has become highly valuable in biomedical investigations due to its user-friendly nature and well-defined biological characteristics. When incubated at 20 °C, newly hatched larvae progress through four distinct larval developmental stages (from L1 to L4) and reach adulthood in only 72 h [5]. Furthermore, *C. elegans* cell lineage remains almost completely invariant from one individual to another. Interestingly, several groups have demonstrated that exposure to chemical or physical stress results in larval growth defects (growth delay, larval arrest) [6], [7], [8]. These features, coupled to extensive literature available, makes *C. elegans* an exceptional model for RT studies, where larval growth can be used to monitor the normal tissue sparing effect of FLASH-RT. Here, we sought to characterize the impact of ionizing radiation in multiple irradiation settings (CDR XR, CDR proton, Ultra-high dose rate proton, CDR electron, and Ultra-high dose rate electron) on *C. elegans* larval development. The introduction of C. elegans as a model for FLASH-RT studies not only overcomes challenges associated with mammalian models but also capitalizes on its small size. The reduced volume for irradiation facilitates the attainment of UHDR conditions, making it adaptable to various machines. Furthermore, the low thickness of the model makes linear energy transfer (LET) probing possible. In this paper, we present the proof of concept that this model serves as a promising and versatile tool for advancing FLASH research in radiotherapy.

## 2 Materials and methods

### 2.1 *C. elegans* strains and maintenance

Methods for the maintenance and handling of *C. elegans* were described by Brenner [9]. Wild type N2 Bristol *C. elegans* were used for all the analysis. The worms were cultivated on nematodes growth medium (NGM) agar plates seeded with *Escherichia coli* strain OP50 at 20 °C. For irradiations, adults *C. elegans* hermaphrodites are bleached to collect embryos. These embryos are then placed on 3.5 cm Petri dishes without food for irradiation.

### 2.2 X-ray irradiation of *C. elegans*

Embryos were irradiated at 0, 10, 20 and 50 Gy with a X-Rad 225XL (Precision X-Ray; USA) at CDR (0.033 Gy/s); 225 kVp; 13.5 mA; 1 mm aluminum filter; 50 cm from the XR source. The dose rate at the sample location is measured using a Farmer® Ionization Chamber (PTW Dosimetry, Germany). Embryos were then collected by washing the plates with M9 buffer and incubated overnight at 20 °C in M9 without food.

### 2.3 Proton beam irradiation of *C. elegans*

Proton irradiations were performed on the ALTAÏS UHDR set-up developed at the LARN laboratory. Briefly, irradiations were performed using a 1 cm^2^ homogeneous broad beam which is defocused using electrostatic lenses and vizualized on a scintillator. The beam homogeneity is analysed using a CCD camera and a ImageJ plugin.The dose rate was measured using a homemade Faraday cup adapted from Thomas et al. [10] especially designed for the current measurement of a low energy proton beam. A pulsing system aligns the beam, using electrostatic plates, for irradiation (in the order of few ms) and the dose is delivered in one pulse. The CDR dosimetry has been previously described by Wéra et al. and Riquier et al. [11], [12]. The embryos were exposed to the 4 MeV proton beam with a dose rate of 1000 Gy/s for UHDR irradiations and 0.033 Gy/s for CDR irradiations. In both conditions, the embryos were exposed to doses of 0, 10, and 20 Gy (LET = 10 keV/µm) (Table 1). After irradiations, embryos were collected and incubated overnight at 20 °C in M9 without food.

**Table 1:** Beamline structure and corresponding mean dose rates for CDR and UHDR irradiations. N/A = Not applicable.

Particle	X-rays	Protons		Electrons	
Machine	X-Rad 225XL, Precision X-Ray	Tandetron accelerator ALTAÏS, HVEE		Mobetron, IntraOp	
Mode	CDR	CDR	UHDR	CDR	UHDR
Energy (MeV)	<0.225	4		9	
LET (keV/µm)	N/A	10		0.2	
Repetition rate (Hz)	continuous	continuous	Monopulse	30	90
Pulse on time (s)	N/A	N/A	N/A	$1.2\times 10^{-6}$	$2\times 10^{-6}$
Pulse off time (s)	N/A	N/A	N/A	$33\times 10^{-3}$	$11\times 10^{-3}$
Average dose rate (Gy/s)	0.033	0.033	1000	0.17	126
Instantaneous dose rate (Gy/s)	0.033	0.033	1000	$4\times 10^{3}$	$6.6\times 10^{5}$
Dose per pulse (Gy)					
10 Gy	N/A	N/A	10	$5.6\times 10^{-3}$	1.35
20 Gy	N/A	N/A	20	$5.6\times 10^{-3}$	1.30
Number of pulses					
10 Gy	N/A	N/A	1	1786	8
20 Gy	N/A	N/A	1	3571	16
Total irradiation time					
10 Gy	5min	5min	10ms	1min	77ms
20 Gy	10min	10min	20ms	2min	165ms
Dosimetric validation	Radiochromic film and ionization chamber	Radiochromic film and PIPS detector	Radiochromic film and Faraday cup	Radiochromic film and ionization chamber	Radiochromic film and ionization chamber

### 2.4 Electron beam irradiation of *C. elegans*

Electron irradiations were performed on a Mobetron (IntraOp, Sunnyvale, CA, USA) electron-beam linear accelerator at the Jules Bordet Institute. This Mobetron was upgraded to operate in both CDR and UHDR and was validated for FLASH irradiations (see validation process for this device described by Moeckli et al. [13]). The embryos were exposed to a 9 MeV electron beam with an average dose rate of 126 Gy/s for UHDR irradiations and 0.17 Gy/s for conventional irradiations. In both conditions, the embryos were exposed to doses of 0, 10, and 20 Gy (LET = 0.2 keV/µm). The CDR irradiations were performed by sending 1.2 µs pulses at 30 Hz and UHDR irradiations were performed by sending 8 or 16 pulses (2 µs width; 90 Hz frequency) of 1.3 Gy respectively (Dose Rate Per Pulse = $6.5\times 10^{5}$ Gy/s). The dose deposition was confirmed for each irradiation using Gafchromic EBT3 radiochromic film (Ashland, KY, USA) calibrated with an Advanced Markus ionization chamber (PTW Dosimetry, Germany) in conventional mode (Table 1). After irradiation, embryos were collected and incubated overnight at 20 °C in M9 without food.

### 2.5 Analysis of the size distribution

The day after the irradiation, L1 synchronized worms were seeded on NGM plate with food (OP50). Microscopic images of the worms were taken 96 h post irradiation. The size calibration was assessed by taking a picture of a 200 µm diameter tungsten wire deposited in the same optical plane than the worms. Pictures were then analyzed using ImageJ as described in [14]. To compare each experiment, length of worms was normalized according to their respective control means and the FLASH Factor (FF) is defined as:

$$\mathrm{FF}={\left(\frac{\mathrm{normalized}\;\;\mathrm{siz}e_{\mathrm{UHDR}}}{\mathrm{normalized}\;\;\mathrm{siz}e_{\mathrm{CONV}}}\right|}_{\mathrm{isodose}}$$

The FF is the reciprocal of the Dose Modification Factor (DMF) therefore, FF = 1/DMF as explained by Farr et al. [15]

### 2.6 Statistical analysis

Statistical analysis was performed using GraphPad Prism (version 10.0.0). The mean value of the worm’s length was compared using a One-Way ANOVA test with Bonferroni correction. All data presented here are individual experimental points (110 to 236 worms per condition). The mean of the experimental points ± standard deviation is presented for each condition.

## 3 Results

### 3.1 Both CONV-XR and CONV-Proton affects *C. elegans* larval development

**Fig. 1:**
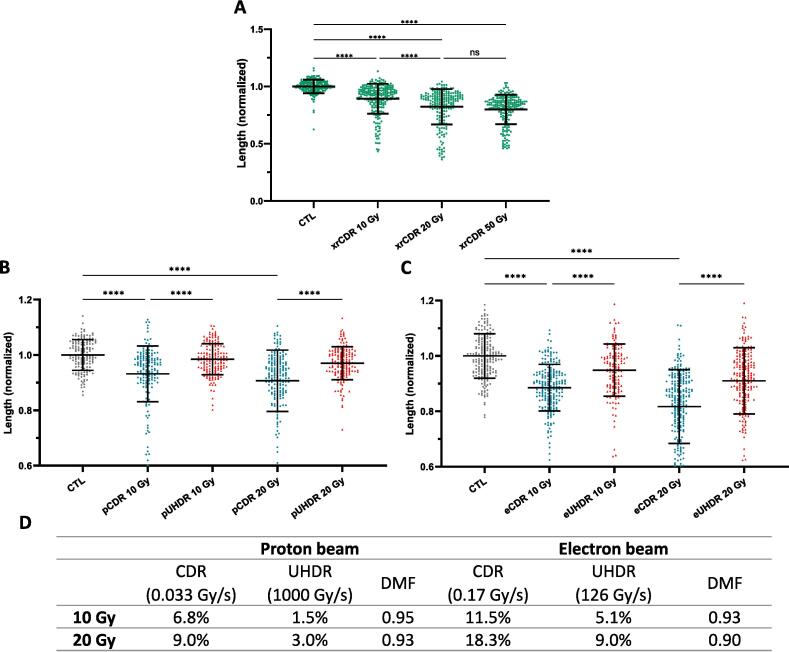
Growth delay 96 h after irradiation induced by CDR-XR (A- green) of C. elegans embryos and comparison of UHDR (red) and CDR (blue) irradiation effect on the growth delay. B. Growth delay after proton irradiations (n = 154–187/point). C. Growth delay after electron irradiations (n = 138–236/point). Worm length following irradiations were normalized by length of unirradiated control samples. **** = p < 0.0001 and ns = not significant (p > 0.05). D. Growth delay observed proportionally to the control conditions using proton or electron beam at CDR and UHDR. The DMF is calculated for each condition according to Farr et al. definition of the FF and the DMF.

We observed a significant reduction in worms’ length for both CDR-XR and CDR-proton at all the tested doses, suggesting a radiation-induced developmental delay (Fig. 1A, B). A dose-dependent behavior was reported until 20 Gy, with no significant difference observed between CDR-XR 20 Gy and higher irradiation doses. Worms irradiated with 10 Gy were 11 % (CDR-RX, 112 ± 13 µm) and 7 % (CDR-proton, 69 ± 8 µm) smaller than non-irradiated control worms (1016 µm). When increasing the dose to 20 Gy, this length difference increased to 18 % (CDR-RX, 184 ± 13 µm) and 9 % (CDR-proton, 91 ± 12 µm) as illustrated on Fig. 1A and B. Given the absence of a significant difference in growth delay between 20 and 50 Gy following CDR X-ray irradiation, irradiations utilizing proton and electron beams were conducted at doses up to 20 Gy.

### 3.2 UHDR proton irradiation limited the growth delay compared to conventional irradiation

After characterizing *C. elegans*’ embryos dose–response to CDR-proton, we increased the dose rate to explore the dose–response at higher dose rates. Notably, for both tested doses, worms were significantly taller following UHDR than CDR-proton irradiation (Fig. 1B). While CDR irradiation led to a 6.8 % and 9.0 % drop in worm length at 10 and 20 Gy, respectively, UHDR exposition only resulted in modest length reductions (1.5 % at 10 Gy; 3.0 % at 20 Gy).

### 3.3 UHDR electron irradiation confirm proton’s results

To validate the aforementioned biological observations (Fig. 1A, B), we reproduced the experiment on a Mobetron® accelerator, a well-known irradiation device able to deliver conventional as well as FLASH electron beams [13]. Results obtained on this irradiation facility showed a consistent reduction in worm length with increasing dose (Fig. 1C), as reported following proton irradiation. Moreover, for both tested doses, worms were significantly taller following UHDR than CDR-electron irradiation, as observed for proton irradiation (Fig. 1C). After 10 Gy, the normalized length reduction observed upon irradiation went from 11.5 % (in CDR-electron irradiation) down to 5.1 % (in UHDR settings). A more pronounced decrease was observed when 20 Gy were delivered: 18.3 % in CDR and 9.0 % in UHDR settings, respectively.

## 4 Discussion

Since its discovery in 2014, the FLASH effect has been studied in a wide variety of animal models and demonstrated using electron, photon and proton beams [16]. In the context of proton FLASH therapy, it is interesting to note that our current understanding is predominantly limited to irradiation in the plateau region and not in the spread-out Bragg peak (SOBP), which is where their main clinical use comes in. While recent studies have expanded our knowledge to include data on the SOBP and pristine Bragg peak protons [17], [18], [19], it is essential to highlight the challenges associated with studying the effect of the Linear Energy Transfer (LET) on the occurrence of FLASH effect. To delve deeper into this aspect, the ALTAÏS accelerator (UNamur; Belgium) is a valuable tool capable to operate at low-energy (≤4 MeV), paving the way to detailed studies of the LET impact on radiation responses. However, the use of low-energy beams, which have short range, necessitates the use of very small animal models (≤200 µm for the 4 MeV proton beam) such as *C. elegans*.

Despite being evolutionary distant from mammals, *C. elegans* has already been shown to be a suitable research tool for cancer research due to many advantages such as its ease to manipulate, size, short lifespan, transparency, and the conservation of biological processes with higher organisms including humans.

At the embryonic stage, *C. elegans* can be considered a stem cell, exhibiting behaviors similar to those found in acute responding tissues. The observed FLASH effect, with a magnitude of approximately 10 %, aligns with findings previously reported in other early responding tissues like the gut in mice and Zebrafish embryos as reported by Kacem et al. [20]. The FLASH effect has been observed for both electron and proton irradiations (Fig. 1B, C) with a DMF of approximately 0.93 at 10 and 20 Gy for both types of beams (Fig. 1D). These findings are consistent with previous calculated DMF for gut, skin and survival in mice, rat skin and zebrafish for doses of the same order of magnitude (10–20 Gy) with both proton and electron beam [16], [20], [21].Interestingly, we found a slightly higher sparing effect when using an electron than a proton beam. This result, which may seem curious, is in line with other studies carried out on ZF ([20], [22], [23]). Nowadays, the reasons for these observations remain to be investigated. Nevertheless, recent studies suggest that part of the answer to this question may lie in radiochemistry of H_2_O_2_ produced during irradiations [20], [24] and the differences between the beam parameters for electrons and protons in both UHDR and CDR conditions.

One other hypothesis which could explain the higher magnitude of FLASH effect observed using electron beam is the higher impact of electron CDR irradiation compared to proton CDR, as depicted in Fig. 1D. This unusual finding could be attributed to differences in CDR values between the electron beam (0.17 Gy/s) and the proton beam (0.033 Gy/s), thus relying on the classical dose rate effect [25], [26] associated to a more detrimental outcome with increasing dose rate due to lack of time for repairing sublethal lesions. Therefore, the FLASH effect observed with electron beam would appear magnified, despite such particles having a lower LET compared to 4 MeV protons. Further investigation to confirm the sparing effect observed at high dose rate is ongoing. For example, irradiations of L1 larvae, young adults and embryos are planned to provide more biological endpoints such as survival and fertility tests in order to observe other manifestations of the FLASH effect on *C. elegans*. Finally, we will soon be able to irradiate at the exact same dose rate both in electrons and protons. Future experiments will therefore compare LET without changing the dose rate between beams.

## 5 Conclusion

This first study of UHDR irradiation on *C. elegans* embryos shows promising results on the usability of this nematode for further FLASH investigations. We have shown that in just 5 days, one can observe a dose-rate sparing effect on *C. elegans* using multiple ionizing radiations. We observed similar DMF to those obtained after UHDR irradiation on usual animal models in the field (mice and zebrafish). These consistent findings pave the way for promising avenues of research with our model, particularly in exploring the intricate effects of LET in hadron FLASH therapy.

## CRediT authorship contribution statement

**Lucas Schoenauen:** Conceptualization, Methodology, Investigation, Formal analysis, Writing – original draft. **François-Xavier Stubbe:** Conceptualization, Methodology. **Dirk Van Gestel:** Writing – review & editing. **Sébastien Penninckx:** Conceptualization, Writing – review & editing. **Anne-Catherine Heuskin:** Supervision, Funding acquisition, Writing – review & editing.

## Declaration of competing interest

The authors declare that they have no known competing financial interests or personal relationships that could have appeared to influence the work reported in this paper.
